# Recreational and Medical Cannabis Legalization and Opioid Prescriptions and Mortality

**DOI:** 10.1001/jamahealthforum.2023.4897

**Published:** 2024-01-19

**Authors:** Hai V. Nguyen, Emma E. McGinty, Shweta Mital, G. Caleb Alexander

**Affiliations:** 1 School of Pharmacy, Memorial University of Newfoundland, St John’s, Newfoundland & Labrador, Canada; 2 Division of Health Policy and Economics, Weill Cornell Medicine, New York, New York; 3 College of Pharmacy, University of Manitoba, Winnipeg, Manitoba, Canada; 4 Center for Drug Safety and Effectiveness, Johns Hopkins Bloomberg School of Public Health, Baltimore, Maryland; 5 Department of Epidemiology, Johns Hopkins Bloomberg School of Public Health, Baltimore, Maryland; 6 Division of General Internal Medicine, Johns Hopkins Medicine, Baltimore, Maryland

## Abstract

**Importance:**

While some have argued that cannabis legalization has helped to reduce opioid-related morbidity and mortality in the US, evidence has been mixed. Moreover, existing studies did not account for biases that could arise when policy effects vary over time or across states or when multiple policies are assessed at the same time, as in the case of recreational and medical cannabis legalization.

**Objective:**

To quantify changes in opioid prescriptions and opioid overdose deaths associated with recreational and medical cannabis legalization in the US.

**Design, Setting, and Participants:**

This quasiexperimental, generalized difference-in-differences analysis used annual state-level data between January 2006 and December 2020 to compare states that legalized recreational or medical cannabis vs those that did not.

**Intervention:**

Recreational and medical cannabis law implementation (proxied by recreational and medical cannabis dispensary openings) between 2006 and 2020 across US states.

**Main Outcomes and Measures:**

Opioid prescription rates per 100 persons and opioid overdose deaths per 100 000 population based on data from the US Centers for Disease Control and Prevention.

**Results:**

Between 2006 and 2020, 13 states legalized recreational cannabis and 23 states legalized medical cannabis. There was no statistically significant association of recreational or medical cannabis laws with opioid prescriptions or overall opioid overdose mortality across the 15-year study period, although the results also suggested a potential reduction in synthetic opioid deaths associated with recreational cannabis laws (4.9 fewer deaths per 100 000 population; 95% CI, −9.49 to −0.30; *P* = .04). Sensitivity analyses excluding state economic indicators, accounting for additional opioid laws and using alternative ways to code treatment dates yielded substantively similar results, suggesting the absence of statistically significant associations between cannabis laws and the outcomes of interest during the full study period.

**Conclusions and Relevance:**

The results of this study suggest that, after accounting for biases due to possible heterogeneous effects and simultaneous assessment of recreational and medical cannabis legalization, the implementation of recreational or medical cannabis laws was not associated with opioid prescriptions or opioid mortality, with the exception of a possible reduction in synthetic opioid deaths associated with recreational cannabis law implementation.

## Key Points

**Question:**

What is the association of recreational and medical cannabis legalization with opioid prescriptions and fatal overdoses in the US?

**Findings:**

In this cohort study using state-level data and a generalized difference-in-differences method that accounted for possible contamination from multiple laws, there was no discernible association found between cannabis laws and opioid prescriptions nor fatal opioid overdose, although the results suggested a potential reduction in synthetic opioid deaths associated with recreational cannabis laws. These results were robust to excluding state economic indicators, accounting for additional opioid laws and using alternative ways to code treatment dates.

**Meaning:**

The study results suggest that recreational and medical cannabis legalization were not associated with significant increases or decreases in opioid prescriptions and fatal overdose with the exception of a possible reduction in synthetic opioid deaths that was associated with recreational cannabis law implementation.

## Teaser

This cohort study examines changes in opioid prescriptions and opioid overdose deaths associated with recreational and medical cannabis legalization in the US.

## Introduction

Despite the efforts of many parties, the opioid epidemic persists in the US, with more individuals experiencing fatal overdoses during 2021 than any year on record.^1^ Continued opioid-related harms have generated interest in potential mechanisms to ameliorate the crisis, including the legalization of cannabis for recreational or medical use. For example, it is theoretically possible that individuals with acute or chronic pain may substitute cannabis for prescription opioids, thereby reducing opioid use and opioid-related harms.^2,3^ Greater availability of cannabis, a substance with a much lower risk profile of overdose than opioids, may also be associated with reduced initiation of prescription or illicit opioids. However, many clinical and economic barriers may prevent the substitution of cannabis for prescription opioids or the translation of such substitution into reduction in downstream harms.^4,5,6,7^ There are also concerns that cannabis may serve as a gateway to illegal drug use.^8^

Empirical work examining the association between cannabis legalization and opioid-related outcomes in the US has yielded mixed conclusions. Some evidence suggests that medical cannabis legalization has been associated with reductions in opioid prescribing,^9,10,11^ and in some cases, opioid-related mortality,^12^ whereas other evidence suggests that medical cannabis legalization was not associated with changes in opioid prescriptions^13^ or has been associated with increased, rather than decreased, opioid-related mortality.^14,15,16,17^ Studies examining the effect of recreational cannabis laws have found reductions in opioid prescriptions associated with recreational cannabis legalization,^18,19,20,21,22^ and evidence on the effects of recreational cannabis laws is mixed, with studies indicating either no association with or an increase in opioid mortality.^14,15,16^

Apart from finding mixed results on opioid overdose deaths, the current literature has several limitations. Most studies use traditional difference-in-differences (DD) analyses to estimate the effects of legalization. Recent advances in the DD literature indicate that estimates of policy effects in these analyses may be contaminated when policy effects vary over time or across states.^23^ Moreover, the bias can be compounded when multiple policies adopted in a staggered fashion are assessed at the same time, as in the case of medical and recreational cannabis laws. While some studies have sought to address the issue of heterogeneous policy effects by conducting stacked regressions^14^ or considering a subset of states or time periods,^15^ to our knowledge, no study has rigorously accounted for biases that could arise from evaluating medical and recreational cannabis law implementation at the same time. Also, most studies examine the associations with opioid prescriptions in specific populations (eg, Medicaid enrollees or individuals with employer insurance). In this study, we quantified the association of recreational and medical cannabis law implementation with opioid prescriptions and mortality in the US using national data and a novel analytic approach that overcame the limitations of previous studies including possible biases due to heterogeneous policy effects and simultaneous evaluation of medical and recreational cannabis laws.^23^

## Methods

### Study Design

This study followed the Strengthening the Reporting of Observational Studies in Epidemiology (STROBE) reporting guidelines. We used publicly available, state-level aggregate data. Thus, ethics approval was not required based on Newfoundland and Labrador’s Health Research Ethics Board guidelines.

Estimating the effects of state cannabis law implementation entails 2 challenges. First, different states adopted these laws at varied points (eTable 1 in Supplement 1). Recent literature, including studies by Callaway and Sant’Anna^24^ and de Chaisemartin and d’Haultfoeuille,^25^ has shown that treatment effects (overall and dynamic effects over time) estimated using traditional DD methods may be biased if the laws are implemented in a staggered fashion and the effects of the laws vary across states or over time (for example, as implementation ramps up).^24,25,26,27,28^ Second, states implemented multiple laws that could be associated with opioid outcomes, including not only recreational and medical cannabis laws but also other laws designed to curb opioid prescriptions and overdose. As a result, estimates of 1 law’s effect could be contaminated by that of others.

To address these challenges, we used a generalized DD design recently developed by de Chaisemartin and d’Haultfoeuille^23^ to test the association between state cannabis laws and opioid-related outcomes. This method was suitable for estimating the effects of a staggered intervention while accounting for other interventions that could be associated with the outcomes of interest. Like other methods, this method involves comparing outcome changes in treated states (states that implemented the law) with changes in outcomes in control states, but it carefully defines and selects valid control states for these comparisons. Specifically, it uses only control states that had not yet implemented the law but had the same other laws as treated states during the baseline (defined as the year before the cannabis law was implemented). Further details on this method, its underlying assumptions, and its advantages vs alternative methods to estimate effects of multiple treatments are provided in the eMethods in Supplement 1.

### Treatment and Control Assignment

A state was considered treated in a period if it had cannabis dispensaries operating during that period (eFigure 1 and eTable 1 in Supplement 1). Dates of dispensary openings were obtained from Mathur and Ruhm.^14^ As outcome data were available at the yearly level, we constructed yearly indicators for recreational and medical cannabis dispensary openings. If the dispensaries were opened during the first half of a year, we coded the indicator as 1 for that year (and subsequent years) and 0 otherwise. We tested the sensitivity of our results to this coding (as described later).

For each treated state with a cannabis law, we identified control states as those that had not yet implemented the cannabis law and had the same confounding opioid laws as the treated state during the year before cannabis law implementation. We considered 3 potentially confounding opioid laws, namely mandatory prescription drug monitoring program laws (that require prescribers to check the prescription drug monitoring program database before prescribing an opioid), Good Samaritan laws (that provide legal protection to individuals calling for help in the event of an overdose), and naloxone access laws (including those allowing standing orders in which prescribers may authorize pharmacists to dispense naloxone without an outside prescription or permitting first responders to carry naloxone).

As an example, Colorado implemented a medical cannabis law in 2010, a Good Samaritan law in 2012, a naloxone access law in 2013, and a recreational cannabis law in 2014. To estimate changes in outcomes associated with recreational cannabis laws in Colorado, this method used the period after 2013 during which there was only 1 legislative change, the recreational cannabis law in 2014, and compared outcome changes in Colorado with outcome changes in control states (eg, Washington) that had not yet implemented recreational cannabis laws but, up to 2013, already had implemented medical cannabis laws and adopted a Good Samaritan law and a naloxone access law like Colorado. The list of control states for each treated state is provided in eTable 2 in the Supplement 1.

### Data Sources, Study Period, and Outcomes

We used publicly available data from the US Centers for Disease Control and Prevention (CDC). Opioid prescription rates were based on the IQVIA Xponent database, which collects data from more than 50 000 retail pharmacies, accounting for more than 90% of all retail prescriptions across the US.^30^ Data on the number of opioid overdose deaths were derived from the National Vital Statistics Multiple Cause of Death files (*International Statistical Classification of Diseases and Related Health Problem *[*ICD*]*, Tenth Revision* underlying cause-of-death codes X40–X44, X60–X64, X85, and Y10–Y14 and multiple cause of death codes T40.1-T40.4) and available from the public-access CDC Wide-Ranging Online Data for Epidemiological Research (CDC-WONDER) online database.^29^ We focused on the period from January 2006 through December 2020 due to the availability of data on opioid prescriptions during that window.

The outcomes of interest were the number of opioid prescriptions (per 100 persons) and number of opioid overdose deaths (per 100 000 population), calculated at state-year level for all 50 US states and Washington, DC. Prescription opioids included buprenorphine (except products to treat opioid use disorder), codeine, fentanyl, hydrocodone, hydromorphone, methadone, morphine, oxycodone, oxymorphone, propoxyphene, tapentadol, and tramadol.^30^

### Statistical Analysis

We implemented the approach of de Chaisemartin and d’Haultfoeuille^23^ using regression analysis. All regressions controlled for 2 state-level economic indicators (ie, state poverty rates and real gross domestic product). All regressions also included state indicators to control for time-invariant state-level characteristics and year indicators to control for secular changes or shocks in outcomes that are common to all states. Detailed descriptions of the variables in the regression are provided in eTable 3 in Supplement 1.

We modeled all outcomes using linear regressions. As the method of de Chaisemartin and d’Haultfoeuille^23^ evaluates only 1 policy at a time, the effects of medical and recreational cannabis laws were estimated in separate regressions. Also, the opioid policies were used only to identify valid control states for the analysis; thus, their effects were not estimated by the regressions. For comparison, we also used traditional DD analyses to examine the association of cannabis law implementation with opioid outcomes. In addition to examining the association of cannabis law implementation with overall opioid overdose mortality, we conducted subgroup analyses in which we assessed these associations with opioid mortality by the type of opioid involved in overdose.

We also conducted additional analyses to examine the robustness of the results. First, we examined the sensitivity of our results to the exclusion of time-varying state-level economic indicators. Second, we coded the treatment exposure indicators as 1 for the whole year if the legalization came into effect anytime during the first 3 quarters of that year (instead of anytime during the first half of the year in the main analysis) and 0 otherwise. Third, we accounted for additional opioid laws, namely the prescription limit laws that restricted the number of days that clinicians dispensed opioids for acute pain and pill mill laws. Fourth, we restricted the analysis period to 2011 to 2020 as the nature of the opioid crisis changed around 2011 to 2012 when prescription opioids started to decline rapidly and as opioid overdose deaths started growing at an accelerated pace around 2014 to 2015 (which was driven by heroin and synthetic opioids). Finally, to assess the potential validity of the parallel trends assumption, we examined the prepolicy trends in treated vs control groups using event study based on the approach of de Chaisemartin and d’Haultfoeuille^23^ and the traditional DD analyses. All analyses were performed with Stata, version 17 (StataCorp). We implemented the method of de Chaisemartin and d’Haultfoeuille^23^ using Stata command *did_multiplegt*. All tests were 2-sided, and a 5% significance level was used.

## Results

A plot of unadjusted differences in outcomes between states with and without recreational or medical cannabis laws against the time since opening of cannabis dispensaries is shown in eFigure 2 in Supplement 1. This plot suggested no association of cannabis law implementation with opioid outcomes in most states.

Table 1 presents the adjusted associations of interest. Each entry in this panel is from a separate regression model. The changes in prescribed opioids associated with recreational cannabis law implementation were not statistically significant (3.08 fewer prescriptions per 100 persons; *P* = .17), with the 95% CI ranging from a decrease of 7.43 prescriptions (or a 10% decrease compared with the annual average of 73.4 prescriptions across all states during the study period) to an increase of 1.27 prescriptions (or a 2% increase). Neither were the changes in opioid overdose mortality (3.05 fewer deaths per 100 000 population; *P* = .24) statistically significant, ranging from a decrease of −8.18 deaths (or a 78% decline compared with the annual average of 10.5 deaths across all states during the study period) to an increase of 2.07 deaths (or a 20% increase). The changes in the outcomes associated with medical cannabis law implementation, while larger in magnitude than those for recreational cannabis law implementation, were also not statistically significant at 3.54 additional prescriptions per 100 persons (95% CI, −1.49 to 8.57; *P* = .17) and 3.09 additional deaths per 100 000 population (95% CI, −0.26 to 6.44; *P* = .07).

**Table 1.** Changes in Opioid Prescriptions and Opioid Deaths Associated With Cannabis Law Implementation in the US From 2006 to 2020^a^

Laws	Change in opioid prescriptions per 100 persons (95% CI)	*P* value	Change in opioid overdose deaths per 100 000 population (95% CI)	*P* value	No.	
					Treated states	Control states^b^
Recreational cannabis	−3.08 (−7.43 to 1.27)	.17	−3.05 (−8.18 to 2.07)	.24	7	22
Medical cannabis	3.54 (−1.49 to 8.57)	.17	3.09 (−0.26 to 6.44)	.07	15	47

^a^ Data are for 2006 to 2020. Regressions were estimated using the method proposed by de Chaisemartin and d’ Haultfoeuille (details provided in the Methods section).^23^ Standard errors were clustered at the state level. The de Chaisemartin and d’Haultfoeuille method estimates coefficients for recreational cannabis laws and medical cannabis laws in separate regressions. It evaluates the estimated effects of only 1 policy (eg, recreational cannabis law) at a time and used other policies (eg, opioid policies) only to identify the comparison states. Thus, the effects of other policies were not estimated. In addition, control variables (such as state poverty rates and real gross domestic products) were included in the analysis, but in such a way that their potential confounding associations with the outcomes were controlled for, but not directly estimated.

^b^ Some of the treated states served as control states for other treated states in the analysis.

The results for opioid prescriptions using the method of de Chaisemartin and d’Haultfoueille^23^ were broadly consistent with those from traditional DD analyses (eTable 4 in Supplement 1). For opioid mortality, the results were also consistent for medical cannabis laws. However, for recreational cannabis laws, the estimates were large, negative, and statistically significant using the traditional DD method, but smaller and no longer statistically significant using the method of de Chaisemartin and d’Haultfoeuille.^23^

**Figure 1.**
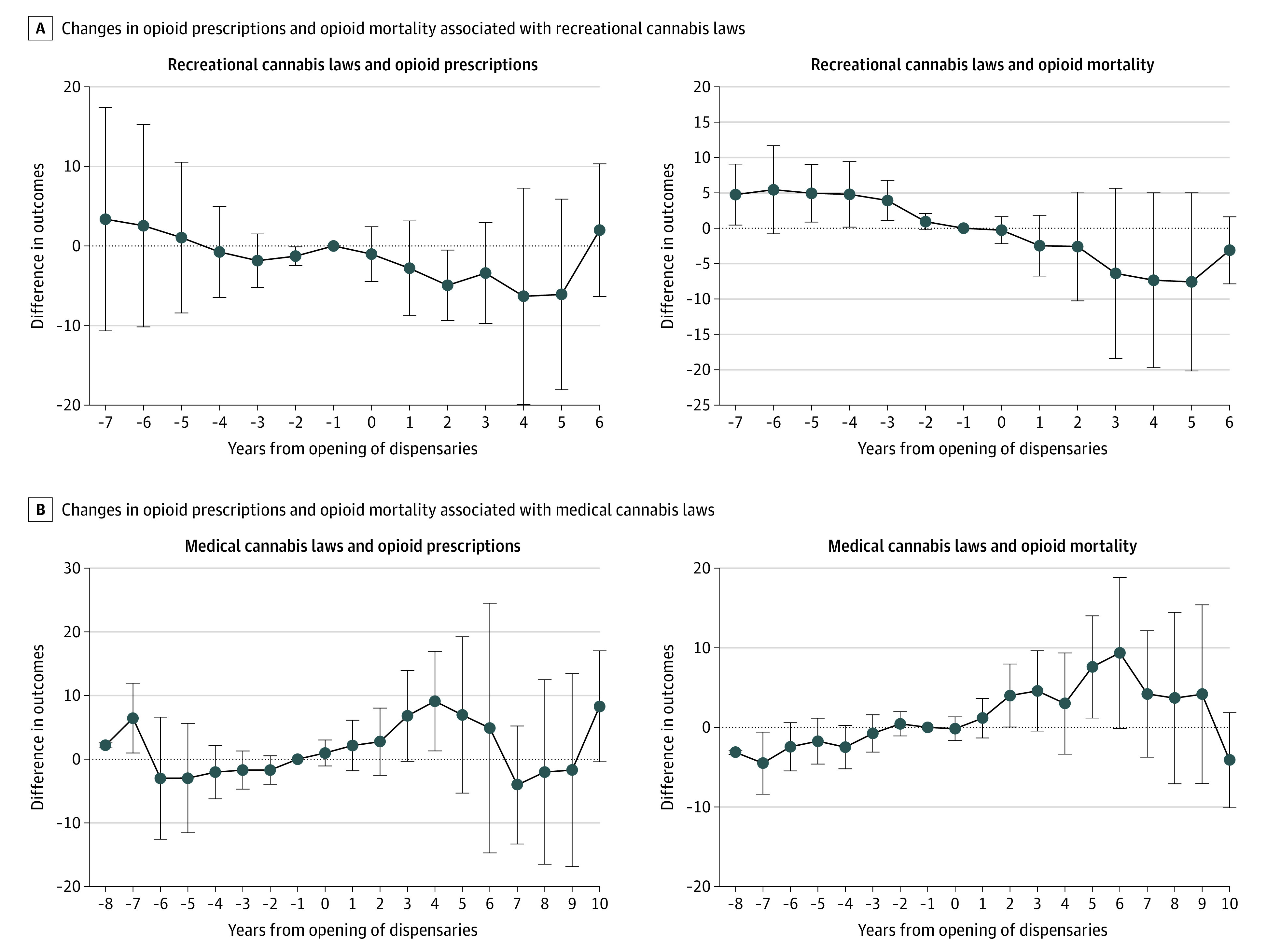
Changes in Opioid Outcomes Over Time Associated With Cannabis Law Implementation Shown are differences in outcome evolution between treated and control states in each year after treated states implemented the cannabis laws (and the associated confidence intervals). Time 0 represents the date when recreational or medical cannabis dispensaries were first opened.

**Figure 2.**
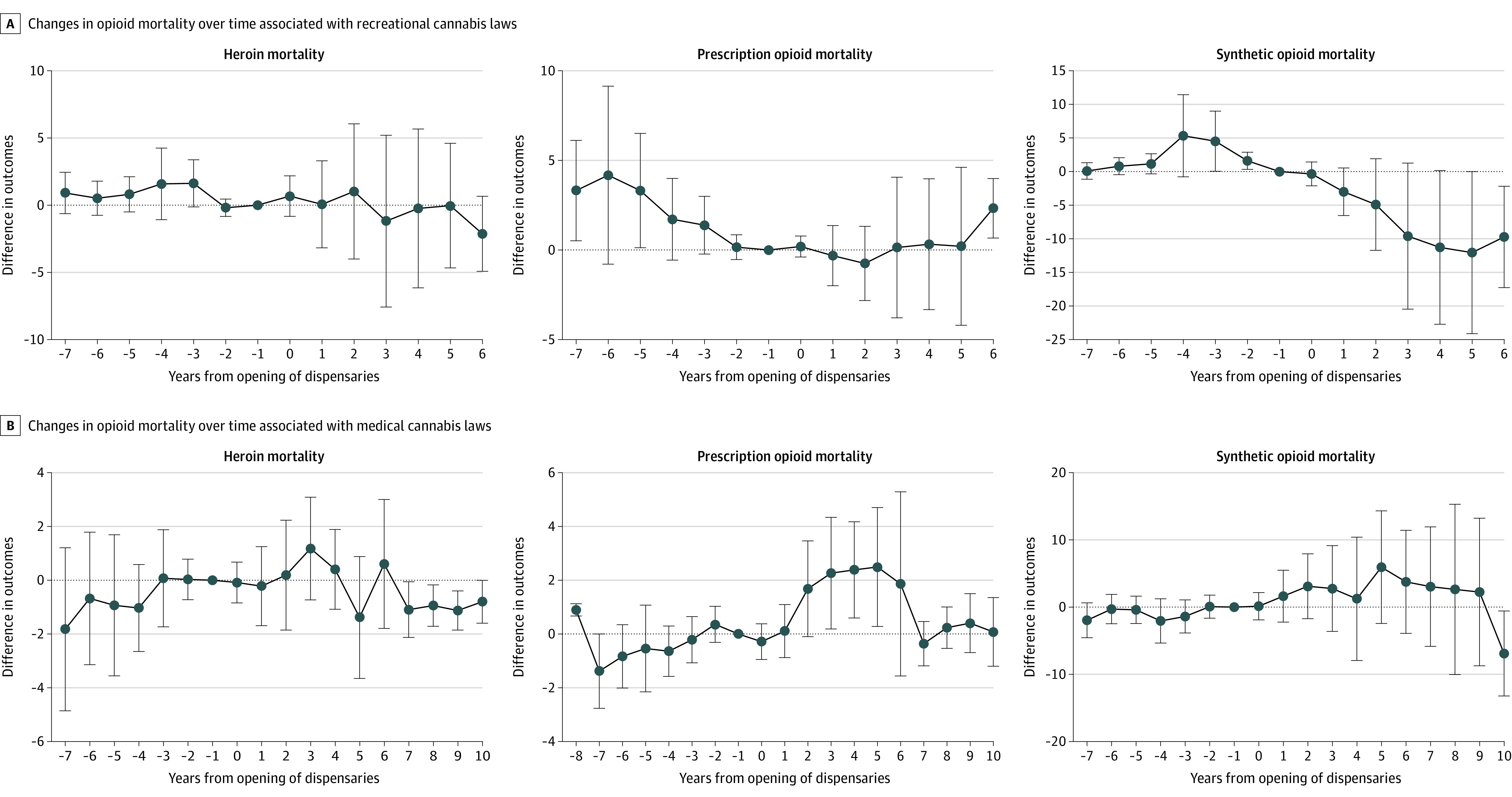
Changes in Opioid Mortality Over Time Associated With Cannabis Law Implementation by Opioid Type Shown are differences in outcome evolution between treated and control states in each year after treated states implemented the cannabis laws (and the associated confidence intervals). Time 0 represents the date when recreational or medical cannabis dispensaries were first opened.

The sensitivity analyses showed that the results for recreational and medical cannabis legalization were robust to the exclusion of time-varying state economic indicators (eTable 5 in Supplement 1). The results were also similar to the main analysis when we coded the treatment exposure variable as 1 for the whole year if the laws were implemented anytime during the first 3 quarters of that year and accounted for additional opioid laws. When we restricted the analysis period to 2011 to 2020, the results were similar for recreational cannabis law (ie, recreational cannabis law implementation was not associated with changes in opioid prescriptions or opioid mortality), but there was a statistically significant increase in opioid prescriptions associated with medical cannabis laws. However, among states that legalized medical cannabis and opened medical cannabis dispensary after 2011, many did so shortly after 2011, making the prepolicy period for these states fairly short for the DD analysis. The event study plots using the approach of de Chaisemartin and d’Haultfoueille^23^ (Figure 1 and Figure 2) and traditional DD analyses (eFigure 3 in Supplement 1) indicated that, except for a slight difference in prepolicy trends between treated and control states for the association between recreational cannabis laws and opioid mortality, the parallel trend assumption was satisfied in our DD analyses.

Table 2 presents the association of law implementation with opioid mortality by the type of opioid overdose. We observed a statistically significant decline in deaths due to synthetic opioids (4.9 fewer deaths; 95% CI, −9.49 to −0.30; *P* = .04) associated with recreational cannabis law implementation. Meanwhile, there were no statistically significant changes in mortality due to heroin or prescriptions opioids associated with cannabis laws. Figure 1 and Figure 2 also show the dynamic changes associated with cannabis law implementation. The null associations of cannabis laws with opioid prescriptions and opioid mortality did not change over time. However, implementation of recreational cannabis laws was associated with non–statistically significant gradual decreases in mortality due to synthetic opioids during the first 5 years of implementation. Meanwhile, there were no clear trends in changes in mortality due to heroin or prescription opioids associated with cannabis laws.

**Table 2.** Analyses by Opioid Type Involved in Opioid Overdose Deaths From 2006 to 2020^a^

Drug	Change in opioid overdose deaths per 100 000 population (95% CI)	*P* value
**Heroin**		
Recreational cannabis laws	0.18 (−2.63 to 2.98)	.90
Medical cannabis laws	0.01 (−0.91 to 0.93)	.98
**Prescription opioids**		
Recreational cannabis laws	0.02 (−1.65 to 1.68)	.98
Medical cannabis laws	1.07 (−0.08 to 2.22)	.07
**Synthetic opioids**		
Recreational cannabis laws	−4.90 (−9.49 to −0.30)	.04
Medical cannabis laws	2.05 (−2.11 to 6.22)	.33

^a^ Data are for 2006 to 2020. Regressions were estimated using the method proposed by de Chaisemartin and d’ Haultfoeuille (details provided in the Methods section).^23^ Standard errors were clustered at the state level. The de Chaisemartin and d’Haultfoeuille method estimates coefficients for recreational cannabis laws and medical cannabis laws in separate regressions. It evaluates the estimated effects of only 1 policy (eg, recreational cannabis law) at a time and used other policies (eg, opioid policies) only to identify the comparison states. Thus, the effects of other policies were not estimated. In addition, control variables (such as state poverty rates and real gross domestic products) were included in the analysis, but in such a way that their potential confounding associations with the outcomes were controlled for, but not directly estimated.

## Discussion

While some have argued that cannabis legalization has helped to reduce opioid-related morbidity and mortality, evidence is mixed, and several studies have been subject to potential bias. We used a recently developed generalized DD approach to quantify the association between recreational and medical cannabis laws and opioid prescriptions and mortality in the US between 2006 and 2020. After accounting for time-varying state-level economic indicators and state opioid laws, we found no evidence that implementation of state recreational or medical cannabis laws was associated with changes in opioid prescriptions or overdose deaths. However, there was a statistically significant reduction in overdose deaths due to synthetic opioids associated with recreational cannabis laws, and this reduction appeared to increase gradually during the first 5 years after law implementation.

These results contrast with recent studies that suggested that recreational and medical cannabis legalization are associated with reductions in opioid prescriptions^21,22^ and medical cannabis legalization is associated with an increase in opioid mortality.^14,15,16,17^ These conflicting findings may arise from many factors, including differences in the study cohorts and periods examined, methods of defining the exposures and outcomes of interest, and empirical approaches used to evaluate these laws.

We did not observe statistically significant reductions in opioid prescriptions or overall opioid mortality associated with cannabis laws in any of the analyses we performed during the entire study period. This result was consistent with reports of physicians’ reluctance to recommend cannabis^4,5^ due to the lack of clinical guidelines and insufficient information on efficacy and adverse effects of cannabis^6^ and high economic costs of switching from opioids to cannabis.^7^ However, there was an increase in overdose deaths involving prescription opioids between years 2 and 4 after medical cannabis law implementation, suggesting potential co-use of cannabis and opioids that was not always under clinical supervision. Meanwhile, our finding indicating a possible association between recreational cannabis laws and reduced synthetic opioid deaths may suggest that users of fentanyl (and other synthetic opioids) switched to recreational cannabis or reduced use of other sedating substances, such as benzodiazepines, that can increase overdose risk when mixed with fentanyl.^17,31^ It is also possible that recreational cannabis laws make cannabis more available and thus reduce initiation of opioid use. However, this finding should be treated cautiously given that the quality of data on specific types of opioid deaths varies across states and over time^32^ and there were fewer treated states in years 2 to 5 in the analysis.

### Limitations

Our study had several limitations. First, our analysis used aggregated data at the state-year level, rendering us unable to observe changes in opioid use within individual patients over time or to examine the estimated effects of laws on subgroups of patients. We were also unable to code legalization dates at a more granular month level. However, our sensitivity analyses indicated that the results were robust to different ways of coding the treatment dates. Second, our intervention date captured only the opening of the first (recreational or medical) cannabis dispensary. We were unable to account for number of dispensaries in a specific state owing to lack of availability of longitudinal dispensary data spanning the study period. Lastly, the study data captured only opioids prescribed in outpatient setting; thus, we were unable to shed light on changes in opioid use in hospital and emergency department settings after cannabis legalization.

## Conclusions

More than 2 decades after the opioid epidemic began in the US, more individuals are dying of opioid overdose than ever before. This ongoing morbidity and mortality has heightened interest in how policy interventions, including those governing recreational and medical cannabis, may intersect with the opioid epidemic. Despite this, evidence to date regarding these matters has been highly mixed. In our analyses accounting for the staggered implementation of cannabis laws in a dynamic clinical and policy environment, we found no evidence that the implementation of recreational or medical cannabis laws was associated with opioid prescriptions or opioid mortality, with the exception of a possible reduction in synthetic opioid deaths associated with recreational cannabis law implementation.

## Notes

Supplement 1. eMethods. Technical details of de Chaisemartin and d’Haultfoeuille’s method eTable 1. Dates of Recreational and Medical Cannabis Legalization and Dispensary Opening across states eTable 2. List of control states eTable 3. Variables in regression analyses eTable 4. Comparison of results using traditional difference-in-differences analyses vs de Chaisemartin and d’Haultfoeuille’s method eTable 5. Sensitivity analyses assessing association between cannabis law implementation and changes in opioid prescribing and opioid deaths in the United States. eFigure 1. Timeline of cannabis legalization and cannabis dispensary openings, 2006-2020 eFigure 2. Unadjusted differences in opioid prescriptions and opioid mortality between states with and without recreational (panels A and B) and medical (panels C and D) cannabis dispensaries in relation to time since opening of dispensaries, 2006-2020 eFigure 3. Event study using traditional difference-in-differences

Supplement 2. Data sharing statement
