# Supplementary material for: Recreational and Medical Cannabis Legalization and Opioid Prescriptions and Mortality
Source: JAMA Health Forum. 2024 Jan 19;5(1):e234897. doi: 10.1001/jamahealthforum.2023.4897 (PMC10799258; doi:10.1001/jamahealthforum.2023.4897)
Supplement: Supplement 1. — eMethods. Technical details of de Chaisemartin and d’Haultfoeuille’s method eTable 1. Dates of Recreational and Medical Cannabis Legalization and Dispensary Opening across states eTable 2. List of control states eTable 3. Variables in regression analyses eTable 4. Comparison of results using traditional difference-in-differences analyses vs de Chaisemartin and d’Haultfoeuille’s method eTable 5. Sensitivity analyses assessing association between cannabis law implementation and changes in opioid prescribing and opioid deaths in the United States. eFigure 1. Timeline of cannabis legalization and cannabis dispensary openings, 2006-2020 eFigure 2. Unadjusted differences in opioid prescriptions and opioid mortality between states with and without recreational (panels A and B) and medical (panels C and D) cannabis dispensaries in relation to time since opening of dispensaries, 2006-2020 eFigure 3. Event study using traditional difference-in-differences [file jamahealthforum-e234897-s001.pdf]

Supplemental Online Content

Nguyen HV, McGinty EE, Mital S, Alexander GC. Recreational and medical cannabis legalization and opioid prescriptions and mortality. *JAMA Health Forum*. 2024;5(1):e234897. doi:10.1001/jamahealthforum.2023.4897

eMethods. Technical details of de Chaisemartin and d'Haultfoeuille's method
eTable 1. Dates of Recreational and Medical Cannabis Legalization and Dispensary Opening across states
eTable 2. List of control states
eTable 3. Variables in regression analyses
eTable 4. Comparison of results using traditional difference-in-differences analyses vs de Chaisemartin and d'Haultfoeuille's method
eTable 5. Sensitivity analyses assessing association between cannabis law implementation and changes in opioid prescribing and opioid deaths in the United States
eFigure 1. Timeline of cannabis legalization and cannabis dispensary openings, 2006-2020
eFigure 2. Unadjusted differences in opioid prescriptions and opioid mortality between states with and without recreational (panels A and B) and medical (panels C and D) cannabis dispensaries in relation to time since opening of dispensaries, 2006-2020
eFigure 3. Event study using traditional difference-in-differences

This supplemental material has been provided by the authors to give readers additional information about their work.

© 2024 Nguyen HV et al. JAMA Health Forum.

eMethods. Technical details of de Chaisemartin and d'Haultfoeuille's method

Our study examines the association between recreational and medical cannabis laws and opioid outcomes. A key challenge to isolating the effects of these laws, however, is that states implemented cannabis and opioid laws in a staggered fashion and the effects of these laws can vary over time and across states. If the effects of medical cannabis and opioid laws on outcomes differ between states that did vs did not implement recreational cannabis laws, these differential effects would not cancel out in standard difference-in-differences analyses and contaminate the effects of recreational cannabis laws (and similarly for medical cannabis laws). In this study, we use the recently developed de Chaisemartin and d'Haultfoeuille's method to overcome this contamination problem.

This method is designed to estimate the effects of a staggered intervention while accounting for other interventions that could affect the outcomes of interest. Like other difference-in-differences analyses, the method compared pre-post changes in outcomes in the treated vs its control states, but it carefully defines and selects valid control states for these comparisons. Specifically, it uses only control states that had not yet implemented the law and had the same other laws as treated states in the baseline (defined as the year before the cannabis law was implemented).

Using recreational cannabis law as an example, this method first identifies control states (for each treated state) as those that had not yet implemented recreational cannabis laws, and at the same time, had the same medical cannabis laws and opioid laws in the year before recreational cannabis law implementation. We considered three potentially confounding opioid laws, namely, mandatory prescription drug monitoring program (PDMP) laws (that require prescribers to check the PDMP database prior to prescribing an opioid), Good Samaritan laws (that provide legal protection to individuals calling for help in the event of an overdose), and naloxone access laws (including those allowing standing orders whereby prescribers may authorize pharmacists to dispense naloxone without an outside prescription or permitting first responders to carry naloxone).

To provide an example of control state selection, Colorado implemented a medical cannabis law in 2010, a Good Samaritan law in 2012, a naloxone access law in 2013, and a recreational cannabis law in 2014. To estimate changes in outcomes associated with recreational cannabis laws in Colorado, this method uses the period after 2013 during which there was only one legislative change – the recreational cannabis law in 2014 – and compares outcome changes in Colorado with outcome changes in control states (e.g., Washington) that had not yet implemented recreational cannabis laws but, up to 2013, already had implemented medical cannabis laws and adopted a Good Samaritan law and a naloxone access law like Colorado. As another example, North Dakota implemented medical cannabis law in 2019 and Good Samaritan and naloxone access laws in 2015. Missouri – which had not implemented medical cannabis laws – and which had also adopted Good Samaritan and naloxone access laws by 2019 could therefore serve as a control state for North Dakota.

After selecting control states, it estimates difference-in-differences regressions (that essentially compare pre-post changes in outcomes in the treated vs its control states) to obtain the policy effects. These policy effects are estimated for each year post policy separately and as a

© 2024 Nguyen HV et al. JAMA Health Forum.

weighted average across the entire post-law period where the weights are proportional to the number of treated states included in calculating each estimate.

We note that the model examines the association of only one policy at a time (recreational or medical cannabis law) with the outcomes; the other policies (e.g., opioid laws) are used only to define the comparison states and thus, their effects are not estimated. Our analysis also included state level control variables (state poverty rates and real gross domestic products) in such a way that their potential confounding effects on the outcomes are controlled for, but not directly estimated. Specifically, the de Chaisemartin and d'Haultfoeuille method first regresses the outcomes on these control variables (and the time fixed effects) separately for the treated and control states, and then uses the residuals from these regressions as the dependent variable to estimate policy effects.

de Chaisemartin and d'Haultfoeuille's method relies on the following assumptions:

i. *Policy effects are heterogeneous*: Our analysis implicitly assumes that effects of recreational and medical cannabis legalization vary over time (and therefore warrants the use of de Chaisemartin and d'Haultfoeuille's approach). This assumption is plausible as it may take some time for opioid users to respond to the change in statute and switch from opioids to cannabis.

ii. *Balanced panel*: de Chaisemartin and d'Haultfoeuille's approach assumes a balanced panel dataset. This assumption is satisfied in our dataset which includes 15 years of data for each of the 51 jurisdictions (50 states + DC) for a total of 765 observations.

iii. *Independent policies across states*: Similar to conventional difference-in-differences, de Chaisemartin and d'Haultfoeuille's approach assumes that outcomes and cannabis laws *across* states be independent.

iv. *Strong exogeneity and parallel trends*: The strong exogeneity assumption requires that implementation of the policies be not determined by past outcomes. This is a common assumption in difference-in-differences literature. The parallel trends assumption requires that, in the absence of legalization, the outcomes between the year prior to opening of dispensaries and the year after these were opened would have evolved similarly in both the treated and control states. To test these assumptions, we compute placebo estimators recommended by de Chaisemartin and D'Haultfoueille. The results shown in Figures 1 and 2 indicate that these assumptions are satisfied in our context. The graphs show that there is no notable trend in differences in outcomes between treated and control states over time, and the vast majority of these differences at each time point are not statistically different from 0.

v. *Effects of cannabis laws appear after opening of cannabis dispensaries:* Our base case analysis assumes that the effects of cannabis laws begin to appear only after cannabis dispensaries become operational.

To our knowledge, de Chaisemartin and d'Haultfoeuille method is the most suited approach to obtain policy effects in our unique context of staggered policy treatment adoption,

© 2024 Nguyen HV et al. JAMA Health Forum.

heterogeneous treatment effects and multiple treatments. While previous studies have used stacked regressions in similar contexts, this method does not account for multiple treatments, nor does it appropriately weight and aggregate treatment effects. Other methods developed to account for staggered policy adoption or multiple treatments, such as those proposed by Goldsmith et al (2022) and Miao et al (2021), also do not conveniently lend themselves to our context: the method by Goldsmith et al (2022) is primarily designed for randomized clinical trial settings with multiple treatment arms while the method by Miao et al (2021) either requires instrumental variables (which are unavailable) or relies on of multiple binary treatments where the assumption that at least half of the confounded treatments have null effects which is unlikely to may not apply in our context.

© 2024 Nguyen HV et al. JAMA Health Forum.

eTable 1: Dates of Recreational and Medical Cannabis Legalization and Dispensary Opening across states

State	Recreational Cannabis Legalization	Medical Cannabis Legalization	Recreational Cannabis Dispensaries	Medical Cannabis Dispensaries	Mandatory PDMP laws	Good Samaritan laws	Naloxone Access laws
Alabama						Jun-15	Jun-15
Alaska	Feb-15	Mar-99	Oct-16	Oct-16	Jul-17	Oct-14	Mar-16
Arizona	Nov-20	Dec-10		Dec-12	Mar-18	Apr-18	Aug-16
Arkansas		Nov-16			Aug-17	Jul-15	Jul-15
California	Nov-16	Nov-96	Jan-18	Jan-04	Oct-18	Jan-13	Jan-08
Colorado	Dec-12	Dec-00	Jan-14	Jun-10		May-12	May-13
Connecticut	Jul-21	Oct-12		Aug-14	Oct-15	Oct-11	Oct-03
Delaware		Jul-11		Jun-15	Mar-12	Aug-13	Aug-14
DC	Feb-15	Jul-10		Jul-13	Jan-21	Mar-13	Mar-13
Florida		Jan-17		Dec-18	Jul-18	Oct-12	Jun-15
Georgia					Jul-18	Apr-14	Apr-14
Hawaii		Jun-00		Aug-17	Jun-18	Jul-15	Jun-16
Idaho						Jul-18	Jul-15
Illinois	Jan-20	Jan-14	Jan-20	Nov-15	Jan-18	Jun-12	Jan-10
Iowa					Jan-19	Jul-16	Apr-15
Indiana						Jul-18	May-16
Kansas							Jul-17
Kentucky					Jul-12	Mar-15	Jun-13
Louisiana		Aug-19		Aug-19	Aug-14	Aug-14	Aug-15
Maine	Jan-17	Dec-99	Oct-20	Mar-11	Jan-17	May-19	Apr-14
Maryland		Jun-14		Jul-17	Jul-18	Oct-14	Oct-13
Massachusetts	Dec-16	Jan-13	Nov-18	Jun-15	Oct-16	Aug-12	Aug-12
Michigan	Dec-18	Dec-08	Dec-19	Jun-18	Jan-18	Jan-17	Oct-14
Minnesota		May-14		Jul-15		Jul-14	May-14
Mississippi					Mar-18	Jul-15	Jul-15
Missouri		Dec-18		Oct-20		Aug-17	Aug-16
Montana	Jan-21	Nov-04		Dec-16		May-17	May-17
Nebraska						Aug-17	May-15
Nevada	Jan-17	Oct-01	Jul-17	Jul-15	Oct-15	Oct-15	Oct-15
New Hampshire		Jul-13		Apr-16	Jan-16	Sep-15	Jun-15
New Jersey	Feb-21	Oct-10		Dec-12	Nov-15	May-13	Jul-13
New Mexico	Jun-21	Jul-07		Jul-09	Jan-17	Jun-07	Apr-01
New York	Mar-21	Jul-14		Jan-16	Aug-13	Sep-11	Apr-06
North Carolina						Apr-13	Apr-13
North Dakota		Apr-17		Mar-19		Aug-15	Aug-15
Ohio		Sep-16		Jan-19	Apr-15	Sep-16	Mar-14
Oklahoma		Aug-18		Oct-18	Nov-15		Nov-13

© 2024 Nguyen HV et al. JAMA Health Forum.

Oregon	Jul-15	Dec-98	Oct-15	Mar-14		Jan-16	Jun-13
Pennsylvania		May-16			Jan-17	Dec-14	Dec-14
Rhode Island		Jan-06		Apr-13	Mar-15	Jun-12	Jun-12
South Carolina					May-17	Jun-17	Jun-15
South Dakota						Jul-17	Jul-16
Tennessee					Apr-13	Jul-15	Jul-14
Texas					Sep-19	Nov-21	Sep-15
Utah		Dec-18		Mar-20	May-17	Mar-14	May-14
Vermont	Jul-18	Jul-04		Jun-13	Nov-13	Jun-13	Jul-13
Virginia	Jul-21			Oct-20	Jul-15	Jul-15	Jul-13
Washington	Dec-12	Dec-98	Jul-14	Jul-11		Jun-10	Jun-10
West Virginia					May-13	Jun-15	May-15
Wisconsin					Apr-17	Apr-14	Apr-14
Wyoming							Jul-17

Note: Policy dates for recreational and medical cannabis laws were sourced from Mathur & Ruhm (2023)[1] and Anderson and Rees (2021)[2]. Policy dates for opioid laws were sourced from Lee et al. (2021)[3]. Mandatory PDMP laws require prescribers to access the PDMP database before prescribing opioids. Good Samaritan laws provide protection to individuals who call for help in case of an overdose event. Naloxone access laws provide protection for administration of opioid antagonists to reverse opioid overdose[3].

© 2024 Nguyen HV et al. JAMA Health Forum.

eTable 2: List of control states

A. Recreational cannabis law analysis

Treated state	Control states
California	DC, Minnesota, Montana
Colorado	California, New Mexico, Rhode Island, Washington
Illinois	Arizona, Connecticut, Delaware, Florida, Hawaii, Maine, Maryland, New Hampshire, New Jersey, New Mexico, New York, Ohio, Rhode Island, Vermont
Massachusetts	Arizona, Connecticut, Delaware, Hawaii, Illinois, Michigan, New Hampshire, New Jersey, New Mexico, New York, Rhode Island, Vermont
Michigan	Arizona, Connecticut, Delaware, Florida, Hawaii, Maine, Maryland, New Hampshire, New Jersey, New Mexico, New York, Ohio, Rhode Island, Vermont
Nevada	Connecticut, Delaware, Massachusetts, New Hampshire, New Jersey, New Mexico, New York, Rhode Island, Vermont
Washington	California, DC, New Jersey, New Mexico, Rhode Island

B. Medical cannabis law analysis

Treated state	Control states
Arizona	Alabama, Alaska, Arkansas, DC, Florida, Georgia, Hawaii, Idaho, Iowa, Indiana, Kansas, Kentucky, Louisiana, Maryland, Michigan, Minnesota, Mississippi, Missouri, Montana, Nebraska, Nevada, New Hampshire, North Carolina, North Dakota, Ohio, Oklahoma, Oregon, Pennsylvania, South Carolina, South Dakota, Tennessee, Texas, Utah, Vermont, Virginia, West Virginia, Wisconsin, Wyoming
Colorado	Alabama, Alaska, Arizona, Arkansas, Delaware, DC, Florida, Georgia, Hawaii, Idaho, Iowa, Indiana, Kansas, Kentucky, Louisiana, Maine, Maryland, Massachusetts, Michigan, Minnesota, Mississippi, Missouri, Montana, Nebraska, Nevada, New Hampshire, New Jersey, North Carolina, North Dakota, Ohio, Oklahoma, Oregon, Pennsylvania, Rhode Island, South Carolina, South Dakota, Tennessee, Texas, Utah, Vermont, Virginia, West Virginia, Wisconsin, Wyoming
Connecticut	Georgia, Illinois, North Carolina, Utah, Wisconsin
DC	Connecticut, Illinois, Massachusetts, North Carolina
Illinois	Alabama, Florida, Georgia, Maryland, North Carolina, Pennsylvania, Utah, West Virginia, Wisconsin
Louisiana	Arkansas, Georgia, Iowa, Kentucky, Mississippi, Pennsylvania, South Carolina, Tennessee, Virginia, West Virginia, Wisconsin
Maine	Alabama, Alaska, Arizona, Arkansas, Delaware, DC, Florida, Georgia, Hawaii, Idaho, Iowa, Indiana, Kansas, Kentucky, Louisiana, Maryland, Massachusetts, Michigan, Minnesota, Mississippi, Missouri, Montana, Nebraska, Nevada, New Hampshire, North Carolina, North Dakota, Ohio, Oklahoma, Oregon, Pennsylvania, Rhode Island, South Carolina, South Dakota, Tennessee, Texas, Utah, Vermont, Virginia, West Virginia, Wisconsin, Wyoming

© 2024 Nguyen HV et al. JAMA Health Forum.

Maryland	Alabama, Arkansas, Florida, Georgia, Hawaii, Iowa, Maryland, Michigan, Mississippi, North Dakota
Massachusetts	Georgia, Illinois, North Carolina, Utah, Wisconsin
Minnesota	Alabama, Florida, Georgia, Maryland, North Carolina, Pennsylvania, Utah, Wisconsin
New Mexico	No controls
New York	Kentucky, West Virginia
North Dakota	Alabama, Missouri, Nebraska, North Carolina, South Dakota
Ohio	Arkansas, Kentucky, Louisiana, Mississippi, Pennsylvania, South Carolina, Tennessee, Utah, Virginia, West Virginia, Wisconsin
Oklahoma	No controls
Oregon	No controls
Rhode Island	Connecticut, Illinois, New York
Utah	Arkansas, Georgia, Iowa, Kentucky, Mississippi, Pennsylvania, South Carolina, Tennessee, Virginia, West Virginia, Wisconsin
Washington	No controls

C. Treated states excluded from analysis

Recreational cannabis law analysis	Medical cannabis law analysis
Excluded due to lack of control	*Excluded due to lack of control*
None	New Mexico
	Oregon
	Oklahoma
	Washington
Excluded as policy implemented before study period	*Excluded as policy implemented before study period*
None	California
*Excluded due to other policies implemented concurrently**	*Excluded due to other policies implemented concurrently**
Alaska (medical cannabis dispensary opening)	Alaska (recreational cannabis dispensary opening)
Oregon (Good Samaritan law)	Delaware (naloxone access laws)
	Florida (mandatory PDMP laws)
	Hawaii (mandatory PDMP laws)
	Michigan (mandatory PDMP laws)
	Montana (Good Samaritan and naloxone access laws)
	Nevada (mandatory PDMP, Good Samaritan and naloxone access laws)
	New Hampshire (mandatory PDMP laws)
	New Jersey (Good Samaritan law)
	Vermont (Good Samaritan law)

*Other concurrent policy implemented in parentheses

© 2024 Nguyen HV et al. JAMA Health Forum.

eTable 3: Variables in regression analyses

Variable	Description	Value level /distribution
Interventions		
Recreational cannabis legalization	Indicator for whether recreational cannabis legalization was in effect in the state	0 = recreational cannabis legalization not in effect<br>1= recreational cannabis legalization in effect
Medical cannabis legalization	Indicator for whether medical cannabis legalization was in effect in the state	0 = medical cannabis legalization not in effect<br>1= medical cannabis legalization in effect
Other policies		
PDMP law	Indicator for whether mandatory PDMP law was in effect in the state	0 = PDMP law not in effect<br>1= PDMP law in effect
Good Samaritan law	Indicator for whether Good Samaritan law was in effect in the state	0 = Good Samaritan law not in effect<br>1= Good Samaritan law in effect
Naloxone access law	Indicator for whether naloxone access law was in effect in the state	0 = naloxone access law not in effect<br>1= naloxone access law in effect
State level economic indicators		
Real GDP	State real gross domestic product	Mean: $327 billion<br>SD: $408 billion
Poverty rate	Percentage of population living below poverty line	Mean: 12.7%<br>SD: 3.5%
Fixed effects		
State fixed effects	Indicator for each state	For each state S:<br>0 = all states except state S<br>1 = state S
Year fixed effects	Indicator for each year	For each year Y:<br>0 = all years except year Y<br>1 = year Y

© 2024 Nguyen HV et al. JAMA Health Forum.

eTable 4: Comparison of results using traditional difference-in-differences analyses vs de Chaisemartin and d'Haultfoeuille's method

	Change in opioid prescriptions per 100 persons (95% confidence intervals)	P value	Change in opioid overdose deaths per 100,000 population (95% confidence intervals)	P value
**Traditional difference-in-differences**				
Recreational cannabis laws	-3.27 (-10.02 to 3.48)	0.34	-5.31 (-8.88 to -1.75)	0.004
Medical cannabis laws	3.11 (-1.36 to 7.57)	0.17	2.23 (-0.61 to 5.07)	0.12
**de Chaisemartin and d'Haultfoeuille**				
Recreational cannabis laws	-3.08 (-7.43 to 1.27)	0.17	-3.05 (-8.18 to 2.07)	0.24
Medical cannabis laws	3.54 (-1.49 to 8.57)	0.17	3.09 (-0.26 to 6.44)	0.07

Note: Data are for 2006-2020. Standard errors are clustered at the state level.

© 2024 Nguyen HV et al. JAMA Health Forum.

eTable 5: Sensitivity analyses assessing association between cannabis law implementation and changes in opioid prescribing and opioid deaths in the United States.

	Change in opioid prescriptions per 100 persons (95% CI)	P value	Change in opioid mortality per 100,000 population (95% CI)	P value
**Excluding controls for state economic indicators**				
Recreational cannabis laws	-3.09 (-7.52 to 1.33)	0.17	-3.40 (-8.57 to 1.77)	0.20
Medical cannabis laws	3.56 (-1.38 to 8.50)	0.16	3.06 (-0.28 to 6.40)	0.07
**Recoding treatment dates of July-Oct as current year**				
Recreational cannabis laws	-3.23 (-7.79 to 1.32)	0.16	-4.72 (-9.43 to -0.02)	0.049
Medical cannabis laws	1.80 (-2.33 to 5.92)	0.39	1.22 (-1.38 to 3.83)	0.36
**Accounting for prescription limits and pill mill laws**				
Recreational cannabis laws	-3.36 (-8.14 to 1.42)	0.17	-2.87 (-8.01 to 2.26)	0.27
Medical cannabis laws	1.54 (-3.89 to 6.97)	0.58	3.31 (-0.99 to 7.61)	0.13
**Time period 2011-2020**				
Recreational cannabis laws	-3.23 (-7.77 to 1.32)	0.16	-2.97 (-8.19 to 2.24)	0.26
Medical cannabis laws	5.69 (0.65 to 10.75)	0.03	3.50 (-0.53 to 7.53)	0.09

Note: Data are for 2006-2020 (unless otherwise stated). All analyses use the method proposed by de Chaisemartin and d'Haultfœuille (details of estimation provided in Methods section). Standard errors are clustered at the state level.

© 2024 Nguyen HV et al. JAMA Health Forum.

eFigure 1: Timeline of cannabis legalization and cannabis dispensary openings, 2006-2020

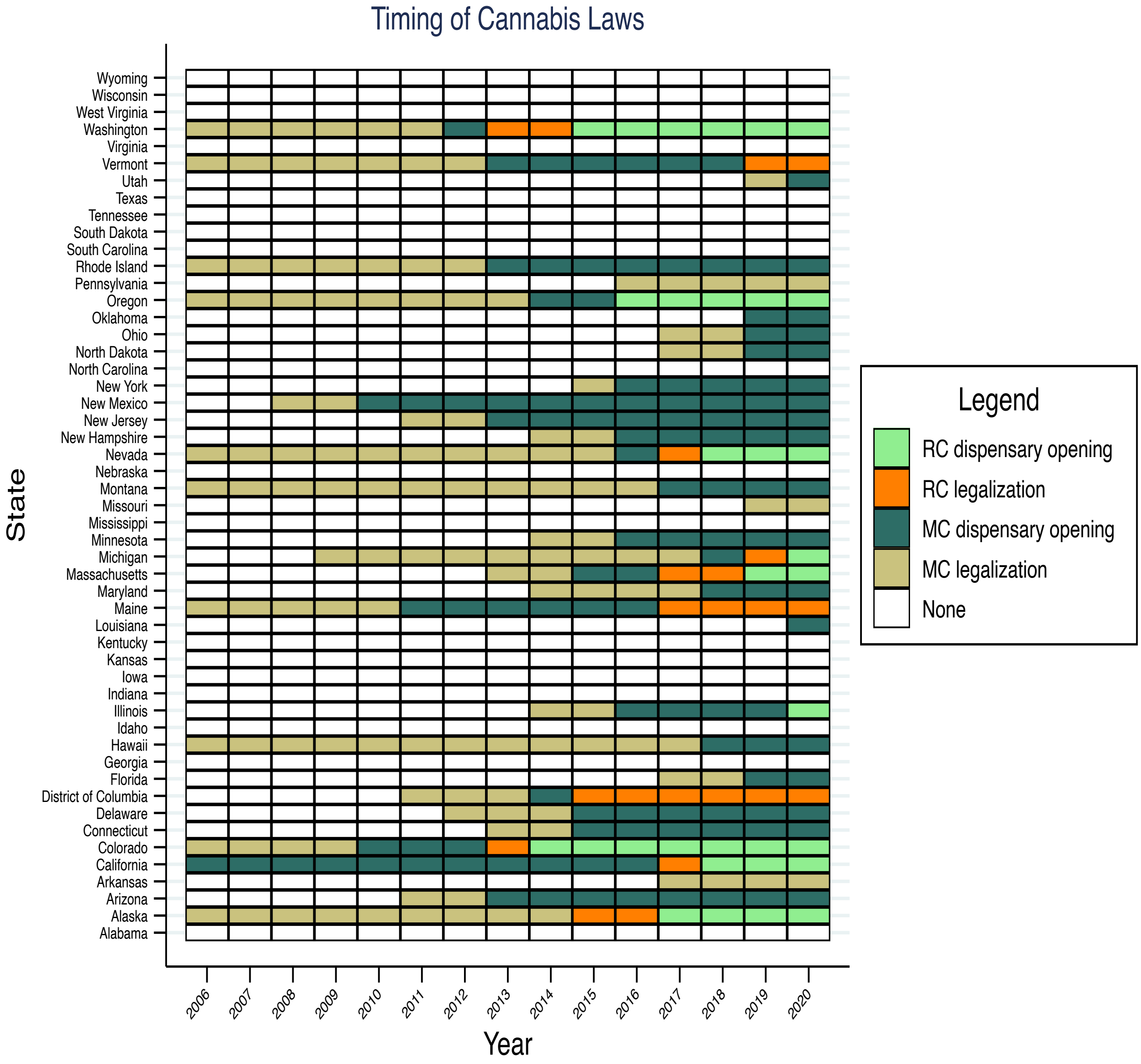

MC: Medical cannabis, RC: Recreational cannabis Some states implemented medical cannabis legalization prior to 2006 (as evident from olive bars in 2006) or opened medical cannabis dispensaries prior to 2006 (as evident from dark green bars in 2006).

© 2024 Nguyen HV et al. JAMA Health Forum.

eFigure 2: Unadjusted differences in opioid prescriptions and opioid mortality between states with and without recreational (Panels A and B) and medical (Panels C and D) cannabis dispensaries in relation to time since opening of dispensaries, 2006-2020.

The figure plots unadjusted differences in outcomes between states with and without recreational or medical cannabis laws, in relation to the time since opening of cannabis dispensaries. For recreational cannabis laws, in most states, the differences in opioid prescribing and opioid mortality in each year after law implementation were similar to the differences before law implementation, suggesting no association of recreational cannabis laws with opioid prescribing and mortality. For medical cannabis laws, compared with the differences before law implementation, the differences after law implementation changed in some states (e.g., lagged increases in Vermont and lagged decreases in Maine for opioid prescriptions and lagged increases for opioid mortality in Delaware). For majority of states, however, the differences changed little after medical cannabis law implementation. Overall, the figure suggests no association of cannabis law implementation with opioid outcomes in most states.

A. Recreational cannabis laws, opioid prescriptions

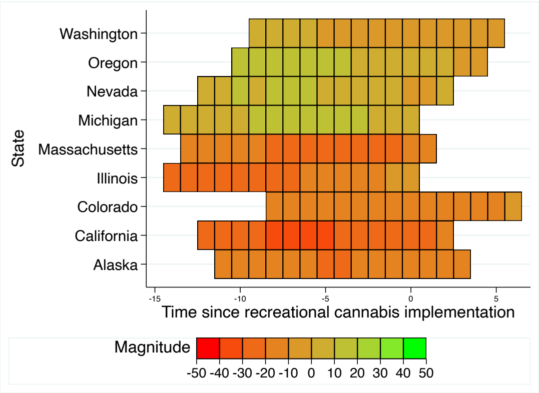

B. Recreational cannabis laws, opioid mortality

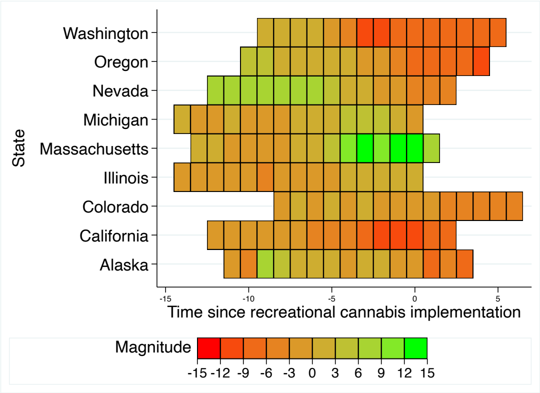

C. Medical cannabis laws, opioid prescriptions.

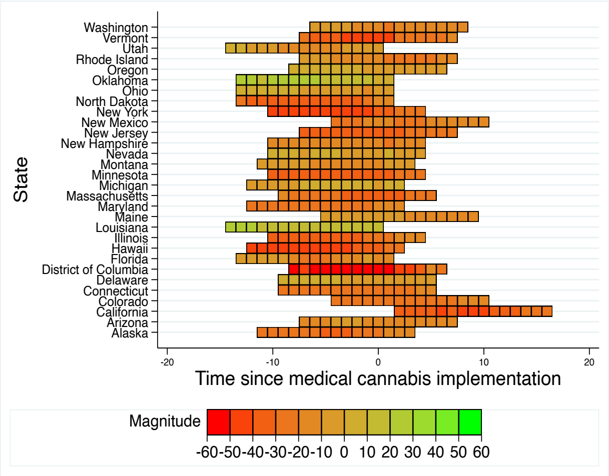

D. Medical cannabis laws, opioid mortality

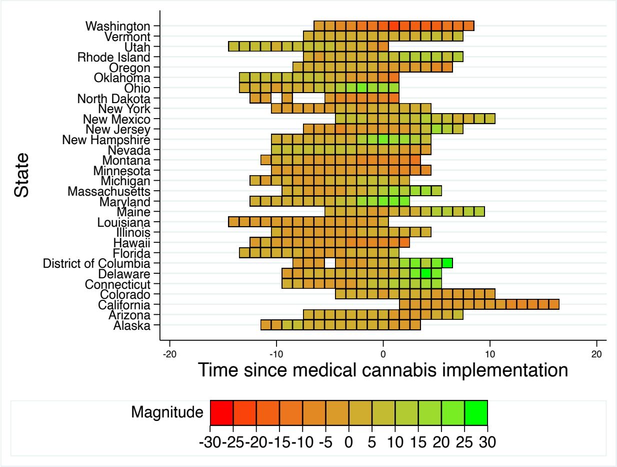

Note: The plots show unadjusted differences in outcomes between states with and without recreational or medical cannabis laws in relation to the time since opening of cannabis dispensaries (time zero). In Panels A and B, states

© 2024 Nguyen HV et al. JAMA Health Forum.

without a cannabis law are those that never opened a recreational cannabis dispensary during our study period while in Panels C and D, these are states that had never opened a medical cannabis dispensary. Each square captures the difference in outcome in a specific year in relation to time zero. Differences in outcomes are calculated separately for each treated state and its control states (where time zero for the control states is the time of policy implementation in the respective treated state). Magnitude of difference is captured by color of the square as described in the figure legend.

© 2024 Nguyen HV et al. JAMA Health Forum.

eFigure 3: Event study using traditional difference-in-differences

A. Event study for association of recreational cannabis law implementation with outcomes

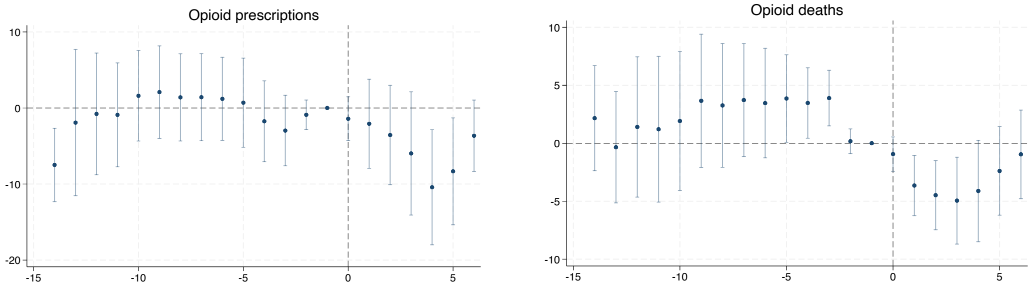

B. Event study for association of medical cannabis law implementation with outcomes

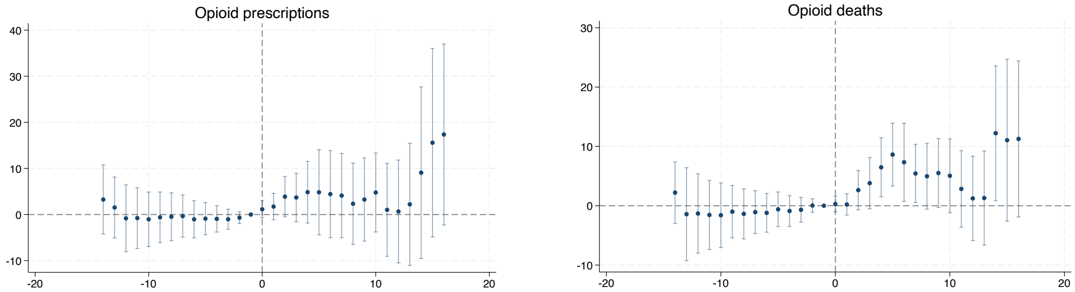

Note: Shown are differences in outcome evolution between treated and control states in each year before and after the cannabis laws. Time zero represents the year in which cannabis law was implemented.

© 2024 Nguyen HV et al. JAMA Health Forum.

References:
1. Mathur NK, Ruhm CJ. Marijuana legalization and opioid deaths. *J Health Econ*. 2023;88:102728.

2. Anderson DM, Rees DI. *The Public Health Effects of Legalizing Marijuana*. National Bureau of Economic Research; 2021.

3. Lee B, Zhao W, Yang KC, Ahn YY, Perry BL. Systematic Evaluation of State Policy Interventions Targeting the US Opioid Epidemic, 2007-2018. *JAMA Netw Open*. 2021;4(2):e2036687. doi:10.1001/jamanetworkopen.2020.36687

© 2024 Nguyen HV et al. JAMA Health Forum.
