# Supplementary material for: Recreational and Medical Cannabis Legalization and Opioid Prescriptions and Mortality
Source: JAMA Health Forum. 2024 Jan 19;5(1):e234897. doi: 10.1001/jamahealthforum.2023.4897 (PMC10799258; doi:10.1001/jamahealthforum.2023.4897)
Supplement: Supplement 2. — Data sharing statement [file jamahealthforum-e234897-s002.pdf]

Data Sharing Statement

Nguyen. Association of Recreational and Medical Cannabis Legalization With Opioid Prescriptions and Mortality. *JAMA Health Forum*. Published January 19, 2024. doi:10.1001/jamahealthforum.2023.4897

Data
Data available: No

Additional Information
Explanation for why data not available: All data used in this study are publicly available from the U.S. Centers for Disease Control and Prevention.
